# Violent Behavior During Psychiatric Inpatient Treatment in a German Prison Hospital

**DOI:** 10.3389/fpsyt.2019.00762

**Published:** 2019-10-31

**Authors:** P. Seidel, N. Konrad, V. Negatsch, D. Dezsö, I. Kogan, U. Gauger, B. Neumann, A. Voulgaris, A. Opitz-Welke

**Affiliations:** ^1^Justizvollzugskrankenhaus, JVA Plötzensee, Berlin, Germany; ^2^Institut für Forensische Psychiatrie, Charité, Berlin, Germany; ^3^Institut für Sexualforschung und Forensische Psychiatrie, Universitätsklinikum Hamburg Eppendorf, Hamburg, Germany

**Keywords:** violent behavior, mental disorder, prison hospital, schizophrenia, age

## Abstract

Violent behavior in correctional facilities is common and differs substantially in type, target, implication, and trigger. Research on frequency and characteristics of violent behavior in correctional facilities and psychiatric hospitals is limited. Results from recent research suggest that comorbidity of severe mental disorder, personality disorder, and diagnosis of substance abuse is related to a higher risk of violent behavior. In the Berlin prison hospital, a database was created to collect data from all violent incidences (n=210) between 1997 and 2006 and between 2010 and 2016. In a retrospective, case-control study, we analyzed specific socioeconomic data and psychiatric diagnosis and compared the group of prisoners with violent behavior with randomly selected prisoners of the same department without violent behavior (n = 210). Diagnosis of schizophrenia, non-German nationality, no use of an interpreter, no children, and no previous sentence remained significantly associated with the dependent variable violent behavior. There were no significant differences regarding age and legal statuses. Practical implications for clinical work are discussed.

## Introduction

Violent behavior is a complex phenomenon linked to biological, psychological, and social factors (1), and it constitutes a common problem in mental health care settings, as well as in correctional facilities. Altogether, there is limited evidence on the prevalence of violent behavior in medical and mental health settings and even less evidence for prison environments. Regarding facilities of community-based mental health care, violent behavior was reported in about 2–7% of all admissions in psychiatric hospitals in Germany (2, 3). Recently, Müller *et al*. reported a moderate increase in violent behavior against staff members in psychiatric inpatient settings between 2008 and 2015, with an average increase in violent incidences of 4% per year (4). A recent meta-analysis, including 23.972 hospitalized psychiatric patients, reported that 17% had at least once acted violently during their hospital stay (5). Staggs *et al*. described no changes from 2007 to 2013 regarding the frequency of violent assaults in U.S. American psychiatric wards (6), but reports from other countries are lacking.

The literature suggests a higher risk of violent behavior in individuals suffering from a severe mental disorder (7–9). Results from a prospective cohort study in Finland (1997) including 12.058 unselected individuals born in 1966 revealed an odds ratio of 3.1 for any criminal offense and an odds ratio of 7.0 for violent offenses in people with schizophrenia (10). Analyzing data of more than 18.000 cases of schizophrenia and other psychosis, Fazel et al. pointed out that the risk for violent behavior was increased compared to the risk of the general population. Furthermore, they described a significant increase in risk for comorbid substance abuse disorder (8). In terms of specific factors for violent behavior in general psychiatry, a history of violent offending, non-adherence to therapy (psychotherapy and/or medication), younger age, male gender, coming from a disadvantaged neighborhood, and recent alcohol misuse were described as risk factors (11) while depressive symptoms and better clinical insight regarding the symptoms were predictors of non-violent behavior (12).

Since the 1960s in Europe and North America, efforts were made to transfer the treatment of individuals suffering from mental disorders from segregated institutions to outpatient treatment facilities placed in the communities. During the last decades, this so-called “deinstitutionalization” was accompanied by a constant reduction of psychiatric beds (13). There is an ongoing discussion of whether the reduction of beds in psychiatric hospitals leads to an increase of severe mentally disordered individuals in prison (14, 15). In a review including 33.588 prisoners in 24 countries, the prevalence of psychotic disorder did not appear to be increasing over time (16). Comparing the level of distress in long- and short-term prisoners in Germany revealed a clinically significant level of depression, paranoid ideation, and psychosis in long-time prisoners (17).

Within the prison system of Berlin, Germany, specialist care is provided for mentally disordered prisoners in the department for psychiatry in the Berlin prison hospital. Admission is possible during every aspect of prisoner life, during remand prison and for the duration of the regular sentence. Typical clinical indications for admission are (exacerbation of) psychosis, suicidal ideation, violent behavior of unclear origin, depression, and adjustment disorders with comorbid personality disorders and substance abuse disorders. Due to the limited size, a waiting list system is implemented to manage the admission process. Also, weekly outpatient treatment is possible directly in the prisons. During the inpatient treatment, a personalized treatment plan includes, e.g., pharmacological treatment and psychotherapy and different options of group therapy including occupational therapy, art therapy, music therapy, addiction therapy, athletic training, and team sports. For severely disordered patients, the possibility of time-limited isolation in specific treatment rooms is available.

In general, in Berlin, male prisoners with a mental disorder are not transferred to a general psychiatric ward outside of the prison system.

If, however, during the trial period, the criminal responsibility of a remand prisoner is found to be diminished, he can be transferred to a forensic psychiatric hospital and, thus, leaves the prison system. Due to regulation through the department of justice, only male prisoners are treated in the department of psychiatry. Female prisoners are treated inside the women prison facility *via* outpatient service or are transferred to a specific forensic psychiatric ward outside of the prison system.

In a current review, the lack of intervention research regarding the prevention of violence in forensic psychiatric settings was identified (18). Regarding prison psychiatry specifically, research on trends and risk factors for violent behavior is rare.

### Aims of Our Study

The first aim of our study was to provide a description of frequency, trends, and pattern of violent behavior in patients of a psychiatric ward in a prison hospital. In a second step, we aim to identify possible risk or protective factors regarding violent behavior in patients of the psychiatric ward in the Berlin prison hospital. Furthermore, we were interested in the changes in the incidence of violent behavior during the last decades.

Our hypothesizes were:

Regarding risk factors for violent behavior research suggests that criminal behavior in the past, younger age, and diagnosis of schizophrenia are risk factors for violent behavior (8, 11, 19–21).

1. We hypothesized that patients with violent behavior were young, had more previous prison sentences, and suffered more often from schizophrenia.
  Due to the often discussed “forensification” of psychiatric patients and the relocation of bed capacity between general and forensic psychiatry (13, 22),
2. We assumed a higher level of violent behavior in the patients of the psychiatric ward of the Berlin Prison Hospital in comparison to known rates from psychiatric inpatients in community hospital care.
3. We expected an increase in patients with a diagnosis of schizophrenia during the study period.
4. We expected that violent behavior in the prison hospital increased during the last 20 years.

## Material and Methods

As part of the routine documentation in German prisons, specific incidents such as violent behavior are reported through a system called “official message” (German: “Dienstliche Meldung”). After 2007, the Berlin prison hospital was no longer an independent unit, but part of the Prison Plötzensee (“JVA Plötzensee”). Consequently, due to administrative changes, the “official message” system was no longer part of medical documentation. From 2010 onwards, new medical files were employed to record patient data. For our study, “official messages” were used to identify patients with violent behavior on the psychiatric ward of the Berlin prison hospital from 1997 to 2006. From 2010 to 2016, we identified violent patients by evaluating medical records. Although a change of the recording system took place during the study period, the basic principles for the assessment of violent behavior remained unchanged.

Between 1997 and 2006, 1,502 “official messages” were documented by the staff members of the psychiatric ward of the Berlin prison hospital. The “official messages” were categorized as “physical violence,” “self-harm,” “verbal violence,” “damage to property,” and “not categorized.” The “not categorized” cases applied when patients offended general prison rules, e.g., behaving noisy, using the telephone without permission, drinking alcohol, or taking drugs. In this study, we only included the cases categorized as “physical violence.”

Altogether, we identified 244 incidents of violent behavior during the period examined, committed by 210 individuals. We compared this group with an equal number of patients who did not demonstrate this behavior during their stay. For the comparison group, we selected the first non-violent individual who was admitted subsequently to each violent individual. For all individuals who generated more than one official message because of violent behavior, we chose the non-violent individual who was admitted directly after the first violent episode as a control. For the actual analysis, the following items were ascertained for both groups: year of the violent act, age of the offender, nationality, using a language interpreter, status of imprisonment (remand or sentenced), previous sentences, self-harming behavior, psychiatric diagnoses, and parenthood/existence of children. The variable “using a language interpreter” was rated as positive when a language interpreter had to be ordered into the prison hospital to translate between the patient and the medical personnel. The variable “parenthood/existence of children” was extracted by analyzing the medical files of the patients. The other variables were rated using both the medical and prison files. Diagnoses were coded using the ICD-10 Manual. For the data from 2010 to 2016, we used a data set that was extracted for research purposes from routine data (23). The data for the same items regarding the period from 1997 to 2006 were extracted from medical files.

The proportion of violent patients of all patients admitted was assessed for each year from 1997 to 2006 and 2010 to 2016. We performed hierarchical linear modeling to test for annual fluctuations in our results. Fisher’s exact test was applied to detect the significance of differences observed between the groups. The impact of all independent variables on the dependent variable “violent behavior” was calculated using a logistic regression model. The subset of all variables that minimize the AIC (Akaike information criterion) was determined by a stepwise elimination procedure to derive a final model. All tests were based on a significance level of p < 0.05. Analyses were performed with the statistical software R, Version 3.5.1. It is important to note that only male prisoners were included in the study.

## Results

**Figure 1:**
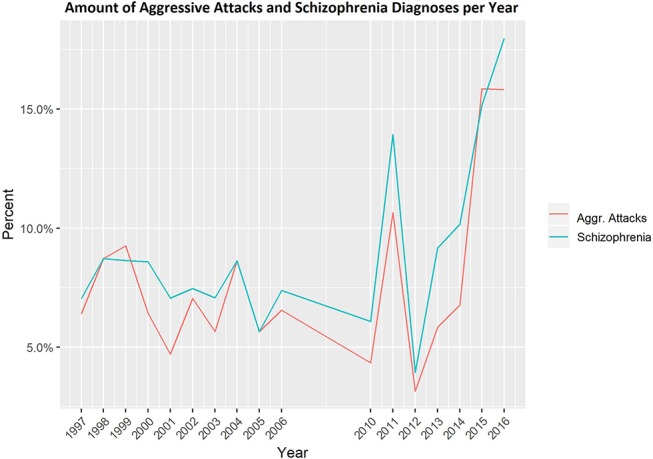
Percentage of admissions of patients with violent behavior per year and of violent patients with schizophrenia per year.

**Table 1** displays the absolute number of patients admitted to the psychiatric ward of the Berlin prison hospital with recorded violent behavior, the number of patients diagnosed with schizophrenia, and the relative share per year. While the percentages of patients with violent behavior in 1997 to 2006 ranged from approximately 4.7 to 9.3%, the percentages of patients exhibiting violent behavior in 2010 to 2016 were subject to more considerable fluctuations (3.2–15.9%). Despite the increase in patients with violent incidences in the last 2 years, test results showed no statistically significant increase over time (p = 0.1543), but the number of individuals in the study group diagnosed with schizophrenia increased significantly (p = 0.0348) (see **Figure 1**).

**Table 1:** Admissions, violent behavior, and diagnosis over time.

Year	Total admissions	Violent behaviorN = 210	% Violent of all admissions/year	N = diagnosis of schizophrenia	% Diagnosis of schizophrenia of study group/yearN = 420
1997	156	10	6.4	11	7.1
1998	149	13	8.7	13	8.7
1999	162	15	9.3	14	8.6
2000	233	15	6.4	20	8.6
2001	255	12	4.7	18	7.1
2002	241	17	7.1	18	7.5
2003	212	12	5.7	15	7.1
2004	197	17	8.6	17	8.6
2005	159	9	5.7	9	5.7
2006	122	8	6.6	9	7.4
2010	115	5	4.4	7	6.1
2011	122	13	10.7	17	13.9
2012	127	4	3.2	5	3.9
2013	120	7	5.8	11	9.2
2014	118	8	6.8	12	10.2
2015	145	23	15.9	22	15.2
2016	139	22	15.8	25	18.0

The univariate analysis of variables associated with violent behavior demonstrated statistically significant results for the items age, previous sentences, nationality, use of an interpreter, children, and diagnosis of a mental disorder (schizophrenia, substance use disorder, and adjustment disorder). While 71.9% of all patients who had displayed violent behavior had no previous convictions, the same only applied to 40.2% of all patients without recorded violent acts (p < 0.001). Having children was also highly significant (p < 0.001), whereas 89.5% of patients with violent behavior did not have children. Also, using the services of an interpreter was significantly lower (p < 0.001) among patients with violent behavior (5.26%) than in patients without recorded incidences (14.3%). Significantly, more patients with a diagnosis of schizophrenia had displayed violent behavior (68.1 *vs*. 47.6%).

All variables outlined above (**Table 2**), including the patient’s age, were entered into a logistic regression model.

**Table 2:** Univariate analysis of variables associated with violent behavior.

	Non-violent group N = 210	Violent group N = 210	p-value
Age (mean +/− SD)	33.6 (+/− 10.5)	31.6 (+/− 9.25)	0.041
Self-harming behavior	29 (13.0%)	26 (12.4%)	0.970
Remand status	50 (23.8%)	49 (23.3%)	1,000
Previous sentence:	126 (59.8%)	59 (28.1%)	<0.001
German nationality	133 (63.3%)	109 (51.9%)	0.023
Using an interpreter	30 (14.3%)	11 (5.26%)	0.003
Children	66 (31.4%)	23 (10.5%)	<0.001
Schizophrenia	100 (47.6%)	143 (68.1%)	<0.001
Alcohol- or drug dependency	22 (10.5%)	9 (4.29%)	0.025
Adjustment disorder	51 (24.3%)	25 (11.9%)	0.002

After a stepwise selection of variables using the Akaike information criterion, the final model was developed (**Table 3**). In this, the variable diagnosis of schizophrenia, non-German nationality, no use of an interpreter, no children, and no previous sentence remained significantly associated with the dependent variable violent behavior (p < 0.05). Note that “age” is not among the independent variables.

**Table 3:** Final logistic regression model of variables associated with violent behavior (final model)*.

	Estimate	Std. error	Adjusted OR (95% CI)	P (Wald´s Test)
Schizophrenia *vs*. no schizophrenia	0.775	0.228	2.17 (1.39, 3.4)	<0.001
German nationality *vs*. no German nationality	−0.633	0.23	0.53 (0.33, 0.84)	<0.007
Interpreter *vs*. no interpreter	−1.260	0.416	0.28 (0.13, 0.64)	0.002
Children *vs*. no children	−1.212	0.295	0.3 (0.17, 0.53)	<0.001
Previous sentence *vs*. no previous sentence	−1.313	0.228	0.3 (0.17, 0.53)	<0.001

*AIC value 486.828, McFadden log likelihood 0.188.

Our results present a rate for patients that demonstrated violent behavior in a prison hospital that ranged from 3.2 to 15.9%. This rate is in the range of rates reported from psychiatric inpatients in community hospital care (2–4). Regarding trends, there was no statistically significant increase in violent behavior in the last 20 years regarding the psychiatric ward of the Berlin prison hospital. Our findings did not support our hypothesis regarding an increase in violent behavior.

Looking for risk and/or protective factors regarding violent behavior, the main findings of our study are that the group of patients that demonstrated violent behavior in the specific setting of a psychiatric ward of a prison hospital differed statistically significant from the non-violent group regarding diagnoses of schizophrenia, nationality, previous sentences, the existence of children, and the use of an interpreter for communication. Interestingly, after logistic regression, there were no group differences for violent behavior regarding age.

Altogether, our findings suggest a strong relationship between suffering from schizophrenia and the frequency of violent incidents but do not support the hypothesis that violent incidents have increased during the study period or are in total more frequent than in community mental health care.

During the last two decades, there is a lively discussion going on whether changes in the provision of mental health care may lead to marginalization and “forensification” of mentally disordered patients. The process of psychiatric deinstitutionalization has changed the structure of psychiatric care during the study period in Germany, in most European countries and the United States (24). Psychiatric beds in community care were closed, and psychiatric care transferred to community-based outpatient service. This process was accompanied by an increase of placements in forensic psychiatric care (25–27), and this finding revived interest in the validity of the “Penrose hypothesis,” which postulates an inverse relationship between the number of psychiatric hospital beds and the size of the prison populations (28). According to Blüml *et al*., the number of psychiatric beds decreased by 12.6% in Germany between 1993 and 2011, and the prison population increased by 14.8%. Nevertheless, the authors argue that statistical analyses point to a more complicated process and that the “Penrose hypothesis” is a univariate simplification of a complex and multifactorial relationship (29). Our findings of an increase in patients with schizophrenia in the group of violent patients may cautiously support the “Penrose hypothesis.”

Due to German law, individuals with mental disorders that committed severe offenses can be admitted directly to forensic psychiatric hospitals instead of prison. It is important to note that bed capacity in forensic psychiatric hospitals increased continuously during our study period (26). Interestingly, in forensic psychiatric hospitals, the literature suggests an increase in violent incidents (21, 22). Maybe, we did not detect a significant increase in violent behavior due to a shift of the most violent subgroup of prisoners with schizophrenia to the care of the local forensic hospital.

Schizophrenia proved to be a statistically significant marker for the patients in the violent group what is in accordance to the international literature on psychosis, substance abuse, and violent behavior (8, 11, 30). Interestingly, rates of violent behavior did not exceed the reported rates from general psychiatry (2–4). Wolf *et al*. recently reported results suggesting that, in specific forensic psychiatric populations, risk factors differ in comparison to general psychiatric populations (31). While in general psychiatric populations, the diagnosis is associated with violent behavior, in forensic psychiatric settings, this is the case regarding gender and previous violent behavior.

There were more patients without a previous sentence in the violent group than in the non-violent group, which contradicted our hypothesis. We hypothesized that there would be a greater percentage of patients with previous sentences in the violent group than in the non-violent group, due to a potentially higher share of patients with antisocial tendencies in the group of individuals with previous sentences and the findings in the literature that criminal problematic behavior in the past is a risk factor for future behavior (8, 19–21, 31). A possible explanation is that the item “previous sentences” may indicate more individual experience in prison settings and, thus, the “shock” of being imprisoned is not as severe as in the group of “first-timers.” It seems understandable that being imprisoned for the first time in combination with a mental disorder is especially traumatizing. After multivariate testing, this item remained statistically significant. As a possible implication for the clinical work, our results suggest that, in a psychiatric prison setting, a detailed medical history should always include the personal criminal record and past experiences with the penal system. To the best of our knowledge, specific studies on this item as a potentially protective factor against violent behavior in prison hospital settings do not exist.

In our sample, most individuals that demonstrated violent behavior had no children of their own, in contrast to the non-violent group. It seems reasonable that the existence of children may be understood as an indirect marker for general social skills such as social competence, the capability of building romantic relationships, and social networking. The international literature on protective factors suggests competencies in these life areas, such as relationships, family, work, and prosocial attitudes (32, 33). Our data supported our hypothesis that the existence of children for an individual may be regarded as a protective factor for violent behavior in a prison hospital setting.

In the violent group, there were significantly more patients of non-German nationality. This variable was significant after logistic regression analyses. Higher incidence of mental disorder, including schizophrenia in migrants, when compared to the resident population, has been reported consistently (34–36). According to current research, reasons for the increased incidence was multifactorial including higher prevalence rates in origin countries, the experience of an elevated level of stress, isolation, exposure to racism, and lower use of medication for psychotic disorders (37–39). In a past analysis regarding the characteristics of psychiatric inpatients in the Berlin prison hospital, there were no hints for an elevated prevalence of psychotic disorders in non-German prisoners (23). The differences in the frequency of violent behavior between German and non-German patients may be attributed to stress-related factors as well as to differences in the acceptance of antipsychotic medication. A limitation of our study regarding the item “mental disorder” was that we did not test for treatment adherence or the specific phase of the psychosis (acute, chronic). The available literature on these topics suggests a relationship between the severity of psychosis and violent or otherwise problematic behavior (11, 12, 40, 41).

Regarding the use of a language interpreter due to the lack of German language skills, this was the case statistically significantly more often in the non-violent group than in the violent group. This result supported our hypothesis that the use of language interpreters could have had a positive influence on violent behavior in our specific patient population. Psychiatric patients with additional deficits in the German language may demonstrate violent behavior more frequently, due to the lack of proper means for communication. The literature on the necessity of a language translator in prison settings concerning problematic behavior is, to our knowledge, minimal (42, 43). The regular interaction through language translators may have positive effects on the patient in the prison environment because; in comparison to the German staff, there is an opportunity for the patient to fully communicate with and through the translator, who is often of the same cultural background.

Regarding the patients in the violent group, it may be possible that the staff was unable to organize interpreters as often or as quick as in the non-violent group, although we did not test for that. Also, maybe due to the initial violent behavior of the patient, a proper appointment with an interpreter was difficult because of specific circumstances (e.g., isolation). Our results suggest a positive influence of language interpreters in a psychiatric prison setting.

International literature suggests that young age is a risk factor for violent behavior in psychiatric patients (44) and the general population. In our prison hospital setting, in the group of violent patients, there were more patients of younger age, but after multivariate analysis, age was not significantly associated with violent behavior. A possible reason for this may be that patients in our prison hospital are less heterogeneous regarding age than in a general psychiatric ward in the community. Still, in our population, patients that showed violent behavior were slightly younger.

Being in remand prison is known to be very stressful for individuals in prison with a significantly higher rate for suicide ideation, self-harm events, and mental distress (45, 46). Our hypothesis that remand prisoners who would be violent more often in our sample did not stand ground after multivariate analysis. In both groups, nearly a fourth of the individuals were remand prisoners. A possible reason could be that patients, once admitted into remand prison, are not always transferred to the prison hospital as soon as possible due to, e.g., lack of capacity. During this critical phase, agitated patients receive treatment in remand prison *via* outpatient psychiatric care and, thus, were not included in our population. It would be interesting to investigate the occurrence of violent behavior in the remand prison system and compare it to the prison hospital and the general prison population. Studies on these issues are missing.

Several limitations must be considered when interpreting our findings. The retrospective design may have led to various biases, such as a variation of the awareness for violent behavior. The fact that reorganization of routine documentation took place during the study period may have caused different rates of reporting violent incidents. Also, our study included only men and excluded women due to the structure of the specific psychiatric ward in the prison hospital in Berlin. Regarding the diagnosis, we did not check for current medication, the severity of symptoms, or the phase of the disorder. Due to incomplete data, we were unable to include the effects of specific personality disorders on violent behavior. High prevalence of personality disorders in prisoners is known, so this could be a focus for future research. Besides, although we covered a rather long time-span of 20 years, the years between 2007 and 2009 were not included due to missing data (see above).

In summary, to the best of our knowledge, this is the first study that explores violent behavior in the setting of a psychiatric ward in a German prison hospital. In our opinion, this is a vital field of research because the professionals in this field are confronted regularly with high-risk populations for violent behavior and because optimization of individual treatment may benefit the long-term outcome for the patient, as well as for the general society. We share the opinion that further research is needed in the area of prison psychiatry, preferably in an international context.

## Data Availability Statement

All datasets generated for this study are included in the article/ supplementary materials.

## Ethics Statement

According to current legal regulation, the study was approved by the local ethic committee at Charité–Universitätsmedizin Berlin.

## Author Contributions

PS, AO-W, AV, and NK designed the study. PS, VN, IK, DD, and AO-W collected the data. PS, AO-W, AV, UG, and BN analyzed and interpreted the data. PS, AV, and AO-W wrote the final draft of the manuscript. PS, AO-W, and NK had full access to all the data in the study and take responsibility for the integrity of the data and the accuracy of data analysis. All authors have contributed to, read, and approved the final version of the manuscript.

## Conflict of Interest

The authors declare that the research was conducted in the absence of any commercial or financial relationships that could be construed as a potential conflict of interest.
