# Corrigendum: Violent Behavior During Psychiatric Inpatient Treatment in a German Prison Hospital

**DOI:** 10.3389/fpsyt.2019.00961

**Published:** 2020-01-17

**Authors:** P. Seidel, N. Konrad, V. Negatsch, D. Dezsö, I. Kogan, U. Gauger, B. Neumann, A. Voulgaris, A. Opitz-Welke

**Affiliations:** ^1^Justizvollzugskrankenhaus, JVA Plötzensee, Berlin, Germany; ^2^Institut für Forensische Psychiatrie, Charité, Berlin, Germany; ^3^Institut für Sexualforschung und Forensische Psychiatrie, Universitätsklinikum Hamburg Eppendorf, Hamburg, Germany

**Keywords:** violent behavior, mental disorder, prison hospital, schizophrenia, age

In the published article, there was an error in affiliation for “D. Dezsö” and “I. Kogan.” Instead of “Justizvollzugskrankenhaus, JVA Plötzensee, Berlin, Germany,” it should be “Institut für Forensische Psychiatrie, Charité, Berlin, Germany.” The affiliations have been updated accordingly.

The authors apologize for this error and state that this does not change the scientific conclusions of the article in any way. The original article has been updated.

